# Scaling Analysis of an Image Encryption Scheme Based on Chaotic Dynamical Systems

**DOI:** 10.3390/e23060672

**Published:** 2021-05-27

**Authors:** L. E. Reyes-López, J. S. Murguía, H. González-Aguilar, M. T. Ramírez-Torres, M. Mejía-Carlos, J. O. Armijo-Correa

**Affiliations:** 1 Insituto de Investigación en Comunicación Óptica, Universidad Autónoma de San Luis Potosí, Álvaro Obregón 64, 78000 San Luis Potosí, Mexico; lerl220591@gmail.com (L.E.R.-L.); marcela.mejia@uaslp.mx (M.M.-C.); 2 Facultad de Ciencias, Universidad Autónoma de San Luis Potosí, Av. Chapultepec 1570, Priv. del Pedregal, 78295 San Luis Potosí, Mexico; hernan@fc.uaslp.mx (H.G.-A.); omararmijo89@gmail.com (J.O.A.-C.); 3 Laboratorio Nacional CI3M, Facultad de Ciencias, Universidad Autónoma de San Luis Potosí, Av. Chapultepec 1570, Priv. del Pedregal, 78295 San Luis Potosí, Mexico; 4 Coordinación Académica Región Altiplano Oeste, Universidad Autónoma de San Luis Potosí, Carretera Salinas-Santo Domingo 200 Salinas, 78600 San Luis Potosí, Mexico; tulio.torres@uaslp.mx

**Keywords:** image encryption system, S-box, two-dimensional multifractal detrended fluctuation analysis

## Abstract

Despite that many image encryption systems based on chaotic or hyperchaotic systems have been proposed to protect different kinds of information, it has been crucial to achieve as much security as possible in such systems. In this sense, we numerically implement a known image encryption system with some variants, making special emphasis when two operations are considered in the scrambling stage. The variants of such an encryption system are based on some hyperchaotic systems, which generated some substitution boxes and the keys of the system. With the aim to have a more complete evaluation, some internal stages of the image encryption scheme have been evaluated by using common statistical tests, and also the scaling behavior of the encrypted images has been calculated by means of a two-dimensional detrended fluctuation analysis (2D-DFA). Our results show that the image encryption systems that include two operations or transformations in the scrambling stage present a better performance than those encryption systems that consider just one operation. In fact, the 2D-DFA approach was more sensitive than some common statistical tests to determine more clearly the impact of multiple operations in the scrambling process, confirming that this scaling method can be used as a perceptual security metric, and it may contribute to having better image encryption systems.

## 1. Introduction

Nowadays, the way in which society communicates has radically changed with the fast development of computers and the internet. In particular, multimedia communication has been gaining momentum in the exchange of information at all social levels. Therefore, in recent years, security and confidentiality have been of considerable interest. Text encryption has been found to be very different from image encryption due to some inherent image characteristics, such as data-rich capacity, high redundancy, and high correlation between adjacent pixels. Due to the demand to have a secure transmission through any means of communication, a great variety of encryption systems has been proposed [1,2,3,4,5].

Chaos theory is used in many fields of science due to its special properties, and cryptography is no exception. Many visual data encryption systems based on chaos theory consider the principle of applying chaotic maps to obtain highly mixing properties, which are similar to cryptographic systems. Encryption systems that involve chaotic systems have been extensively studied due to the large number of properties they present such as ergodicity, pseudo-randomness, and sensitivity to initial conditions, among others. These properties are analogous to the confusion and diffusion stages, which a general encryption system requires. In fact, for an image encryption system to be secure, it must have confusion and diffusion properties [6,7]. The confusion mechanism rearranges the pixel values, while the diffusion mechanism changes the values of each image pixel. To obtain a higher security level, the confusion and diffusion process can be repeated many times [8]. Then, the chaotic systems take a fundamental role to implement new encryption systems, where the system’s performance would be very good against any attack [1,3].

Moreover, with the aim to add more security, some image chaotic encryption algorithms have been included or considered additional stages such as a disturb process at the pixel level. One such example is based on the ZigZag transformation [3,4,5,9]. In [7], another image encryption scheme, with an improvement in security issues that considers a block scrambling and a modified zigzag transformation, has been implemented before encryption, and a key generator based on an enhanced logistic–tent map. Mansouri and Wang [10] presented an encryption system with a new Sine chaotic maps generator, where one-dimensional chaotic maps are used as seed maps to produce new chaotic maps. In fact, these authors consider a one preprocessing scheme on the plain image using different operations. Based on a ZigZag transformation and a three-dimensional logistic chaotic map, the authors of [11] present an encryption system, where at first an scrambling pixel position is considered, and then how the logistic map can be used to diffuse the pixel values in an image. Ahmad and Hwang [12] present a new image encryption system based on chaotic maps and affine transformation with provides a higher key space and removes correlation between adjacent pixels via random chaotic sequences. A new technique of image protection is presented in [1], which decomposes an image into bit-planes by means of XOR-operations between the scrambled images and chaotic map matrix, then the encrypted image is obtained. Karawia [13] presents an algorithm for multiple images using the two-dimensional economic map to get the combination of mixed images elements. As the size of the key space is huge, the latter approach is secure to many different attacks.

Obviously, there are more similar image encryption systems, and one of their typical characteristic is that multiple operations in the scrambling stage are considered, but also with certain advantages or disadvantages when more operations are included in the encryption process. For instance, the process will be more complex and the execution time will be affected, but its security is increased. In this case, it is necessary to find a balance between the security of the encryption system and the processing time. In addition, a few attempts have been made to establish how many operations or transformations are required in an image encryption system, or if an evaluation of internal stages of the complete encryption process may be helpful in the design of image encryption systems. This work is devoted to enhance the safety of image encryption algorithms, and reveal weaknesses in such algorithms. In this sense, we consider the encryption system in [3] and some variants that modify the scrambling stage of the encryption system. In this stage, the original image goes through a process where initially a ZigZag transform is used to get a distorted image. After that, a sorting scramble algorithm or the use of a substitution box (S-box) is applied to the latter image. This allows us to have higher levels of security in the image encryption content, compared to other systems. In particular, in the encryption variants, we combine a ZigZag transform with a S-box, because the S-box substitutes the information content and provides the diffusion properties while maintaining high entropy levels [14]. Despite the chaotic encryption systems presenting a good performance, this study is devoted to measuring the impact of the scrambling process on the quality of the encrypted images and to making an assessment of some stages in the encryption process to see if we can have a stronger encryption system. In addition, to consider some common statistical tests in the assessment, we make usage of the two-dimensional Detrended Fluctuation Analysis (2D-DFA), a tool that can characterize and reveal weaknesses of the content of the encrypted images, where a correlation degree between the surface pixels is obtained. This method has been used to measure the entropy of noisy or textured encrypted images [15], which present values close to unity for this kind of content. It has been also used to measure the similarity between two images [16], where the 2D-DFA value will depend on the content of the processed images, and the similarity degree of the difference of the respective values. Besides, the 2D-DFA complemented the results with some statistical tests to analyze the encrypted image content.

The paper is organized in the following way. In Section 2, a concise presentation of the main elements used in the image encryption systems is given. In Section 3, the image encryption systems and their variants are described, whereas Section 4 contains the results obtained by applying some statistical tests and the two-dimensional DFA technique to the images in different encryption stages. Section 5 is devoted to discuss our main findings, a comparison with some existing works, and some limitations of our proposal. Finally, the conclusions are drawn in Section 6.

## 2. Preliminaries

### 2.1. Hyperchaotic Systems

In this section, we briefly present the two hyperchaotic systems considered in this work. These kinds of systems, despite their simplicity, exhibit more complex dynamics than chaotic systems. They have received wide coverage in different areas of mathematics, physics, and engineering, among others [17,18,19]. The existence of the hyperchaos is verified by checking that there are at least two positive Lyapunov exponents [18]. Besides, according to the Kaplan–Yorke conjecture [20], the Lyapunov dimension ($d_{L}$) of any system in the hyperchaotic regime should be $3<d_{L}<4$.

#### 2.1.1. Hyperchaotic Lorenz System

The hyperchaotic dynamics of Lorenz’s system is modeled by the set of differential equations [17]:

(1) $$\begin{array}{c} \begin{array}{cc} {\dot{x}}^{\left(1\right)} & =x^{\left(2\right)}-x^{\left(1\right)},\\ {\dot{x}}^{\left(2\right)} & =28x^{\left(1\right)}-x^{\left(2\right)}-x^{\left(1\right)}x^{\left(3\right)}+x^{\left(4\right)},\\ {\dot{x}}^{\left(3\right)} & =x^{\left(1\right)}x^{\left(2\right)}-\frac{8}{3}x^{\left(3\right)},\\ {\dot{x}}^{\left(4\right)} & =-5x^{\left(1\right)}, \end{array} \end{array}$$

**Figure 1:**
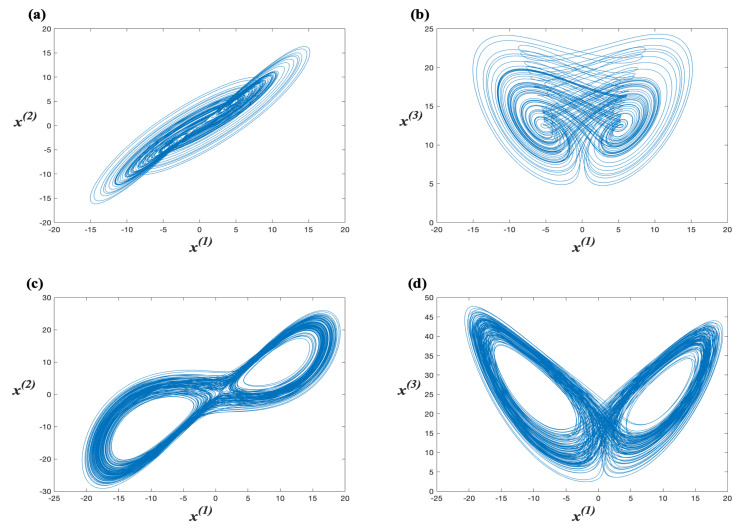
The hyperchaotic attractors of Lorenz’s system projected on the planes (**a**) $x^{\left(1\right)}-x^{\left(2\right)}$ and (**b**) $x^{\left(1\right)}-x^{\left(3\right)}$. The hyperchaotic attractors of Chen’s system projected on the planes (**c**) $x^{\left(1\right)}-x^{\left(2\right)}$ and (**d**) $x^{\left(1\right)}-x^{\left(3\right)}$.

This system is hyperchaotic with Lyapunov exponents $\lambda_{1}=0.38$, $\lambda_{2}=0.41$, $\lambda_{3}=0.00$, $\lambda_{4}=-14.37$, and the Lyapunov dimension is $d_{L}=3.055$, this system is in the hyperchaotic regime. The hyperchaotic attractors generated by Lorenz’s system projected onto the planes $x^{\left(1\right)}-x^{\left(2\right)}$ and $x^{\left(1\right)}-x^{\left(3\right)}$, are shown in Figure 1a,b, respectively.

#### 2.1.2. Hyperchaotic Chen System

We also consider the four-dimensional hyperchaotic system based on Chen’s system as defined in [19,21,22]:

(2) $$\begin{array}{c} \begin{array}{cc} {\dot{x}}^{\left(1\right)} & =36(x^{\left(2\right)}-x^{\left(1\right)}),\\ {\dot{x}}^{\left(2\right)} & =28x^{\left(2\right)}-x^{\left(1\right)}\left(x^{\left(3\right)}-16\right)-x^{\left(4\right)},\\ {\dot{x}}^{\left(3\right)} & =x^{\left(1\right)}x^{\left(2\right)}-3x^{\left(1\right)},\\ {\dot{x}}^{\left(4\right)} & =x^{\left(1\right)}+0.5. \end{array} \end{array}$$

As the Lyapunov exponents are $\lambda_{1}=1.627$, $\lambda_{2}=0.060$, $\lambda_{3}=0.000$, $\lambda_{4}=-12.684$, and the Lyapunov dimension is $d_{L}=3.133$, this system is in the hyperchaotic regime. The hyperchaotic attractors generated by Chen’s system projected onto the planes $x^{\left(1\right)}-x^{\left(2\right)}$ and $x^{\left(1\right)}-x^{\left(3\right)}$, are shown in Figure 1c,d, respectively.

### 2.2. ZigZag Transformation

**Figure 2:**
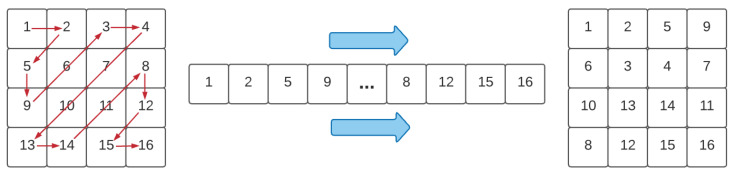
A standard ZigZag transform scheme.

One way to scramble image pixels is to use a ZigZag operation or transform [23]. This operation is usually performed to confuse the elements of the respective matrix data of a plain image. It can reduce the high correlation among image pixels to increase the security level of some encryption systems. To perform the ZigZag transform to the data matrix corresponds to sequentially read the elements of the matrix in a “Z” shape, followed by sequential saving within a data vector, which is reshaped in a certain way into a two-dimensional matrix. Figure 2 shows a standard ZigZag operation [23]. Obviously, there are other ways to implement a different version of the ZigZag transform to avoid that some element positions do not change. For instance, in [3], some improved ZigZag transformations have been considered with change of the scan order of the element positions.

#### 2.2.1. Substitution Box (S-Box) Generation

To shuffle the information of any image, some articles have considered a substitution table known as substitution box (S-box), which is a nonlinear key component in block ciphers of encryption systems [2,14]. Recall that any S-box makes the statistical relationship between the ciphertext and the key as difficult as possible. In this work, we considered the hyperchaotic approach in [2] to generate two particular S-boxes based on the hyperchaotic systems of Lorenz and Chen, whose dynamics are very well modeled by the set of differential equations of (1)–(2), respectively. A reason to choose these hyperchaotic systems is that a persistent scaling behavior was observed in the fourth state of these systems, which may be useful in this kind of applications, see the works in [21,22].

The mechanism to generate the S-box is as follows:

1. Select four initial conditions such that any system of (1) or (2) presents a hyperchaotic behavior, and obtain a state vector $\left(x_{i}^{\left(1\right)},x_{i}^{\left(2\right)},x_{i}^{\left(3\right)},x_{i}^{\left(4\right)}\right)$, for i=0,…,255, corresponding to the normalized state vector of any system of (1) or (2).
2. Multiply the state vector of step 1 by a factor of $10^{8}$ to obtain a new vector $w_{i}=\left({\bar{x}}_{i}^{\left(1\right)},{\bar{x}}_{i}^{\left(2\right)},{\bar{x}}_{i}^{\left(3\right)},{\bar{x}}_{i}^{\left(4\right)}\right)$, for i=0,…,255.
3. Generate a new sequence $S=\{s_{i}\}$, with $s_{i}=\pi_{p+1}\left(w_{i}\right)$, where $\pi_{k}$ is the projection function in the component *k*, and $p={\bar{x}}_{i}^{\left(4\right)}\mathrm{mod}\;3$.
4. Apply a permutation σ of the values {1,…,255} to sequence *S* such that $s_{\sigma (k-1)}<s_{\sigma (k)}$ for k=1,…,255.
5. Generate the S-box Sb={σ(0),σ(1),σ(2),⋯,σ(255)}.

The respective two S-boxes obtained with this scheme are shown in Table A1 and Table A2 of the Appendix A, which are in the conventional representation format.

### 2.3. Two-Dimensional Detrending Fluctuation Analysis

The two-dimensional detrended fluctuation analysis (2D-DFA) algorithm was proposed by Gu and Zhou [24]. A modified and improved version of the 2D-DFA has been used by Vargas-Olmos et al. [15] to analyze encrypted images, as it has been a flexible and efficient method to measure the quality of the encrypted image content. This procedure consists of the following steps by taking into account that an image *I* of size M×N is considered as a surface and denoted by a matrix X(i,j), where the number of rows and columns is represented by i=1,2,…,M and j=1,2,…,N, respectively.

1. Divide the surface X(i,j) into $M_{s}\times N_{s}$ disjoint square windows of the same size s×s, where $M_{s}=\left\lfloor M/s\right\rfloor$ and $N_{s}=\left\lfloor N/s\right\rfloor$. Each window can be denoted by $X_{m,n}$ such that $X_{m,n}\left(i,j\right)=X\left(i+l_{1},j+l_{2}\right)$ for 1≤i,j≤s, where $l_{1}=\left(m-1\right)s$ and $l_{2}=\left(n-1\right)s$.
2. Compute the cumulative sum for each window $X_{m,n}$, positioned by *m* and *n*, as
  $$P_{m,n}\left(i,j\right)=\sum_{k_{1}=1}^{i}\sum_{k_{2}=1}^{j}\left(X_{m,n}\left(k_{1},k_{2}\right)-\langle X_{m,n}\left(k_{1},k_{2}\right)\rangle \right),$$
  where $\langle X_{m,n}\left(k_{1},k_{2}\right)\rangle$ is the average of the sub-image $X_{m,n}$, for 1≤i, j≤s.
3. Determine the trend of the obtained sub-image by fitting the set of data to the plane ${\tilde{P}}_{m,n}\left(i,j\right)=ai+bj+c$, where *a*, *b*, and *c* are parameters which are estimated using the least square method. Subsequently, one calculates the local variances associated to each sub-image $P_{m,n}$ as
  (4) $$F^{2}\left(m,n,s\right)=\frac{1}{s^{2}}\sum_{i=1}^{s}\sum_{i=1}^{s}{\left[P_{m,n}\left(i,j\right)-{\tilde{P}}_{m,n}\left(i,j\right)\right]}^{2}.$$
4. Next, averaging over all sub-images, the overall detrended fluctuation is obtained as
  (5) $$F_{2}\left(s\right)={\left(\frac{1}{M_{s}N_{s}}\sum_{m=1}^{M_{s}}\sum_{n=1}^{N_{s}}F^{2}\left(m,n,s\right)\right)}^{1/2}.$$

This procedure is repeated for a broad range of segment lengths *s*, considering the range 6≤s≤min(M,N)/4. In order to assess a fractal scaling property of the pixelated surface, the fluctuation function $F_{2}\left(s\right)$ should display a power law scaling

(6) $$F_{2}\left(s\right)∼s^{\alpha },$$

where α is called the scaling fluctuation exponent. This scaling exponent can be found as the slope of a double logarithmic plot of $F_{2}$ as a function of *s*, and it is a measure of the degree of correlation among the pixels of the surface. As is pointed out in [15], the fluctuation scaling exponent can be used as an appropriate and objective measure of the quality of encryption algorithms. When the α exponent of the encrypted image is close to 1, then it is supposed that the encryption system is secure from the perceptual point of view. Furthermore, in [16], it is is established that the visual quality of the final encrypted image will be better if the scaling exponent α of the final encrypted image is closer to that of the carrier image.

## 3. Encryption System Model

### 3.1. Encryption System

**Figure 3:**
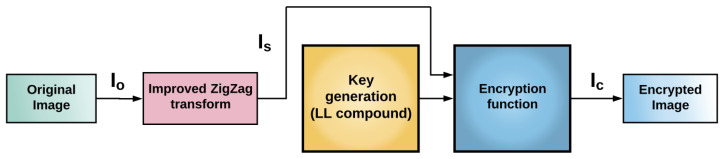
Schematic diagram of the encryption system proposed by Xingyuan et al. [3]. At first, an improved ZigZag transform is applied to the original image ($I_{O}$) resulting in an image $I_{S}$. The latter image and the generated key *K* are the input to the encryption function obtaining an encrypted image $I_{C}$.

In this work, we consider the encryption system employed in [3], which is based on an improved ZigZag transform and a compound of dynamical chaotic systems. The general structure of such an encryption process is shown in Figure 3. This scheme consists of three parts: (1) of an improved ZigZag transform and the chaotic Lü system to scramble the original image pixels, which were complemented by a sorting scramble algorithm and (2) the chaotic Lü system and chaotic logistic map (LL compound) to generate a secure key. (3) Finally, an adjacent-side XOR method is used to complete the image encryption scheme.

### 3.2. Modified Encryption System

**Figure 4:**
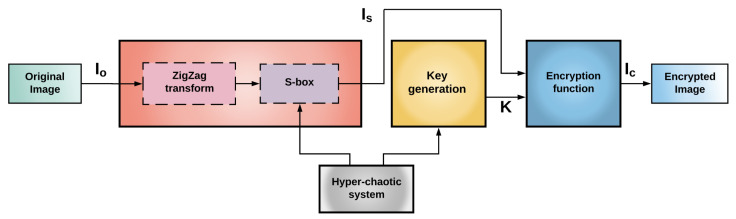
Block diagram of our proposed encryption system. An image $I_{S}$ is obtained after the standard ZigZag, and the S-box procedures are applied to the original image $I_{O}$. The image $I_{S}$ and the generated key *K* are the input to the encryption function resulting in an encrypted image $I_{C}$.

Similarly to the encryption system used by Xingyuan et al. [3], Figure 4 depicts a schematic diagram of the steps involved in our proposal to generate a modified image encryption system, where an S-box and the key generation are based on a hyperchaotic system. Basically, there are two main differences to compute some encryption stages or transformations in the complete encryption system. The first main difference of the previous encryption system is the way to compute the image $I_{S}$ from the original image $I_{O}$. In this modification, we just apply one ZigZag transformation to the image $I_{O}$ and after that an S-box is applied to obtain $I_{S}$. The other difference is the way to carry out the key generation, which takes advantage of the process to compute the S-box, see Section 2.2.1. The main processes of this proposal are described in detail in the following.

Consider an original image $I_{o}$ (or plain-text image) of dimensions M×N, where *M* and *N* are the number of rows and columns, respectively.

Then, proceed with the following steps.

**Step 1**  Apply the scrambling block to the plain text image $I_{O}$, which consists of the application of the standard ZigZag transformation to the image $I_{O}$ and followed by the generated S-box, as is described in Section 2.2.1, to obtain the image $I_{S}$.
**Step 2**  The key generation process is carried out by means of the following approach.
  1. Choose four initial conditions such that any system of (1) or (2) presents a hyperchaotic behavior, depending on which system is used in the S-box generation, and obtain a state vector $\left(x_{i}^{\left(1\right)},x_{i}^{\left(2\right)},x_{i}^{\left(3\right)},x_{i}^{\left(4\right)}\right)$, for i=0,…,n≫1, corresponding to the state vector of the considered hyperchaotic system.
  2. Convert each state vector of step 1 into a new state vector of integer values, $\left({\bar{x}}_{i}^{\left(1\right)},{\bar{x}}_{i}^{\left(2\right)},{\bar{x}}_{i}^{\left(3\right)},{\bar{x}}_{i}^{\left(4\right)}\right)$, where ${\bar{x}}_{i}^{\left(j\right)}=\mathrm{fix}\left(x_{i}^{\left(j\right)}\times 10^{8}\right)$, for j=1,…,4, and the function fix(x) rounds the *x* value to the nearest integer toward zero.
  3. Compute two new vectors: $\left(k_{1}^{1},k_{2}^{1},\ldots ,k_{n}^{1}\right)$ and $\left(k_{1}^{2},k_{2}^{2},\ldots ,k_{n}^{2}\right)$, where
    (7) $$\begin{array}{cc} k_{i}^{1} & =\left(C{\left({\left({\bar{x}}_{i}^{\left(1\right)}\right)}^{2}+{\left({\bar{x}}_{i}^{\left(2\right)}\right)}^{2}+{\left({\bar{x}}_{i}^{\left(3\right)}\right)}^{2}\right)}^{1/2}+\lambda \right)\;\mathrm{mod}\;256, \end{array}$$
    (8) $$\begin{array}{cc} k_{i}^{2} & ={\left(C{\bar{x}}_{i}^{\left(4\right)}+\lambda \right)}^{2}\mathrm{mod}\;256, \end{array}$$
    where i=0,…,n, and *C* and λ are control parameters.
  4. Generate the keys by means of $K_{i}=k_{i}^{1}⨁k_{i}^{2}$, with i=0,…,n, and the symbol ⨁ represents the exclusive OR operation bit-by-bit.
**Step 3**  The encryption function comprises two parts: The first part is called the confusion stage, which is described as
  • For encryption stage: If $K_{i}\;\mathrm{mod}\;2=0$, then $A_{i}=k_{i}^{1}⨁\left(\left(k_{i}^{1}+I_{s_{i}}\right)\;\mathrm{mod}\;256\right)$, otherwise, $A_{i}=k_{i}^{2}⨁\left(\left(k_{i}^{2}+I_{s_{i}}\right)\;\mathrm{mod}\;256\right)$.
  • For decryption stage: $K_{i}\;\mathrm{mod}\;2=0$, then $I_{s_{i}}=k_{i}^{1}⨁\left(\left(A_{i}-k_{i}^{2}\right)\;\mathrm{mod}\;256\right)$, otherwise, then $I_{s_{i}}=k_{i}^{2}⨁\left(\left(A_{i}-k_{i}^{1}\right)\mathrm{mod}\;256\right)$.
  The second part of the encryption function, called the diffusion stage, is described by the following equations:
  (9) $$\begin{array}{cc} U_{i} & =A_{i}\left(\left\lfloor x_{i}^{\left(4\right)}\times 256\right\rfloor \;\mathrm{mod}\;256\right),\\ I_{c} & =\left\{\begin{array}{c} I_{c_{1}}=U_{1}⨁\varphi ,\\ I_{c_{i+1}}=I_{c_{i}}⨁U_{i+1}, \end{array}\right. \end{array}$$
  where $x_{i}^{\left(4\right)}$ is the value of the *i*-th iteration of the fourth state of the hyperchaotic system. $I_{c}$ in (9) is the final result of the encryption system. The encryption key can be represented by an array of six elements: *key* = $\left(x_{0}^{\left(1\right)},x_{0}^{\left(2\right)},x_{0}^{\left(3\right)},x_{0}^{\left(4\right)},\varphi ,\lambda \right)$. The first four elements of the key, $x_{0}^{\left(1\right)}$, $x_{0}^{\left(2\right)}$, $x_{0}^{\left(3\right)}$, $x_{0}^{\left(4\right)}$, correspond to the initial conditions of the hyperchaotic dynamical system, whereas φ and λ are control parameters of the encryption system.
  In order to improve the sensitivity of the cipher, the value of φ should not be too small. In such a case, some adjustments are proposed. For example, considering that the original image to be encrypted is $I_{O}=\left\{p\left(i,j\right)\right\}$, where p(i,j) is the value of the pixel at position (i,j), then, the following values are calculated:
  (10) $$\begin{array}{cc} H_{1} & =\overset{M}{\underset{i=1}{⨁}}\overset{N}{\underset{j=1}{⨁}}p\left(i,j\right),\\ H_{2} & =\sum_{i=1}^{M}\sum_{j=1}^{N}p\left(i,j\right)\mathrm{mod}\;256, \end{array}$$
  where $H_{1}$ is the exclusive OR operation of all the grayscale values in the original image $I_{O}$, whereas $H_{2}$ is the sum of all pixels modulo 256. With the $H_{1}$ and $H_{2}$ values, then $\varphi^{\prime }=K+H_{1}H_{2}$ and $\lambda^{\prime }=\left(\lambda +H_{1}H_{2}\right)\mathrm{mod}\;256$ are calculated. The $\varphi^{\prime }$ and $\lambda^{\prime }$ values will be replaced by the values of φ and λ, respectively.

## 4. Results and Performance Analysis

To measure the impact of the scrambling process on the quality and the robustness of the image encryption system, some common statistical tests have considered such as the histogram analysis, the correlation among the adjacent pixels, the entropy, and the 2D-DFA metric. In order to make a comparison, we consider the image encryption systems discussed in Section 3.1: the system used in [3], called $E_{1}$; our modification with the hyperchaotic Lorenz and Chen system, denominated as $E_{2}$ and $E_{3}$, respectively; and one more system based on our modification, called $E_{4}$, which considers the S-box from [2].

The complete numerical implementation of the image encryption algorithms were performed under the MATLAB R2017b software on a Mac mini with Intel i3 quad-core, CPU 3.6 GHz, and 8 GB RAM memory. In addition, all the hyperchaotic systems considered here were simulated numerically with the classical fourth-order Runge–Kutta algorithm.

### 4.1. Database of the Images

**Figure 5:**
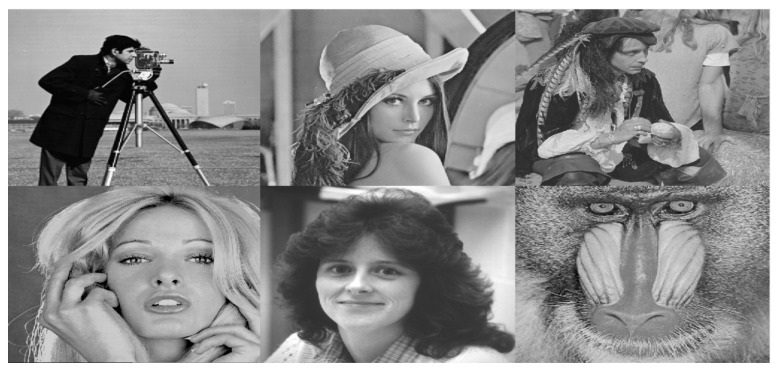
The image dataset considered in this work. Original images ($I_{O}$) of size 512×512, each image is numbered 1 to 6 from left to right and top to bottom.

With the aim to evaluate the performance of our proposal, a representative test bank of images with different characteristics is considered. In particular, a total of six gray-level images were used in this study. All of them have dimensions of 512×512 pixels, and they have been chosen because they are widely used as standard test images in the field of image processing. These original images $I_{O}$ are shown in Figure 5, which are freely available at http://www.imageprocessingplace.com/root_files_V3/image_databases.htm (accessed on 15 April 2021).

### 4.2. Histogram Analysis

Histogram analysis is an important statistical feature of the images, which is generally used to evaluate the performance of image encryption systems. An image histogram shows how pixels in an image are distributed by plotting the number of pixels at each color intensity level. If the histogram of an encrypted image has a uniform distribution, then the encryption system is able to hide the redundancy of original image [3,16].

**Figure 6:**
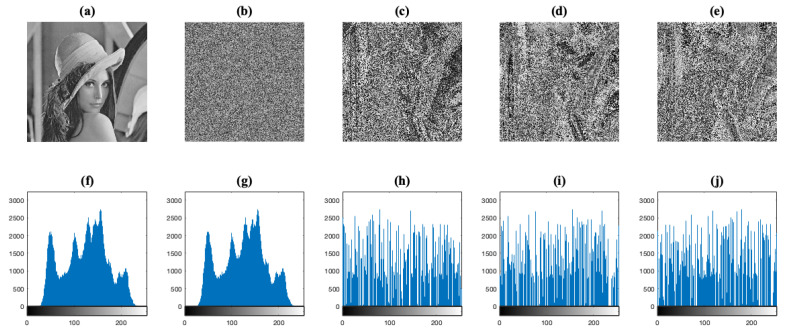
Histogram analysis for the Lena test image. (**a**) The plain image $I_{O}$. (**b**–**e**) The images $I_{S}$ considering the image encryption systems $E_{1}$, $E_{2}$, $E_{3}$, and $E_{4}$, respectively. (**f**–**j**) The corresponding histograms of images (**a**–**e**).

We calculate the histograms for all gray-level images of the image database, and their respective images $I_{S}$ and $I_{C}$ considering the four image encryption systems. As an example, the histograms of the Lena test image and its respective images $I_{S}$ are shown in Figure 6. We can observe in Figure 6c,d acceptable levels of confusion in the visual form of the data when an S-box is considered in the encryption systems $E_{2}$, $E_{3}$, and $E_{4}$, respectively. It is clear that in these cases we cannot achieve a complete unintelligible form as the $E_{1}$ system achieved, see Figure 6e. We will see that the previous results with another encryption stage or transformation, then we can achieve a better unintelligible form. In the bottom row of the same figure, their respective histograms images are displayed. One can see that there is no difference between the histograms of images $I_{O}$ and $I_{S}$ of $E_{1}$ system, Figure 6f,g, respectively, whereas the rest of them do not present a similarity between the histogram of the original image $I_{O}$ with its respective histograms of images $I_{S}$.

**Figure 7:**
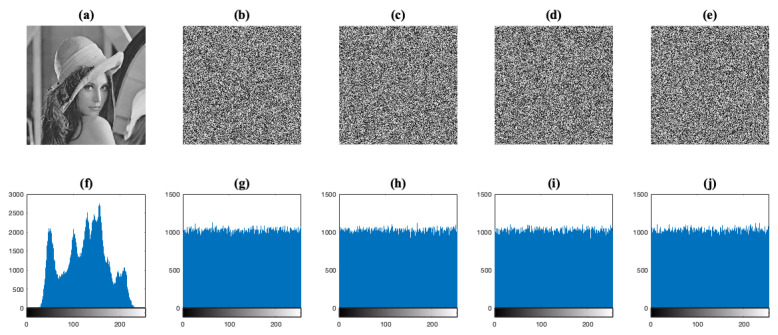
Histogram analysis for the Lena test image. (**a**) The plain image $I_{O}$. (**b**–**e**) The encrypted images $I_{C}$ with the image encryption systems $E_{1}$, $E_{2}$, $E_{3}$, and $E_{4}$, respectively. (**f**–**j**) The corresponding histograms of images (**a**–**e**).

Similarly to the previous case, the histograms of the Lena test image and its encrypted images $I_{C}$ are shown in Figure 7. In these cases, the encrypted images $I_{C}$ achieved an unintelligible form. Moreover, one can see that the histograms of the encrypted images are uniformly distributed and significantly different from the respective histogram of the Lena test image. Therefore, all of the image encryption schemes can make statistical analysis unfeasible to some extent.

With the aim to verify that the encrypted image histogram follows a uniform distribution, and as is pointed out in [25], we consider the chi-square test using

$$\chi^{2}=\sum_{j=1}^{256}\frac{{\left(O_{j}-\mu_{j}\right)}^{2}}{\mu_{j}},$$

where $O_{j}$ and $\mu_{j}$ are the observed and the expected occurrence frequencies of each pixel (0–255), respectively. Using a level of significance of α=0.05, the *p*-values for each of the encrypted images are shown in Table 1, where the null hypothesis is not rejected if the *p*-value is greater than α=0.05. Therefore, it is concluded that the histograms present a uniform distribution for this level of significance.

### 4.3. Correlation between Adjacent Pixels

It is known that plain images usually present a high correlation between their adjacent pixels, a feature that exposes their security making it vulnerable to statistical attacks [3]. If the coefficient is close to 0, it suggests that there is no linear correlation or a weak linear correlation. Therefore, a well-designed encryption system should not present a high correlation in the horizontal, vertical, and diagonal directions. To show that the encrypted image is independent of the test plain image, we calculate the correlation coefficient between the adjacent pixels of both images using

(11) $$\begin{array}{cc} r_{xy} & =\frac{\mathrm{cov}(x,y)}{\sqrt{D(x)}\sqrt{D(y)}}, \end{array}$$

where

$$\begin{array}{cc} \mathrm{cov}(x,y) & =\frac{1}{K}\sum_{i=1}^{K}\left(x_{i}-E\left(x\right)\right)\left(y_{i}-E\left(y\right)\right),\\ D(x)= & \frac{1}{K}\sum_{i+1}^{K}{\left(x_{i}-E\left(x\right)\right)}^{2},\;D\left(y\right)=\frac{1}{K}\sum_{i+1}^{K}{\left(y_{i}-E\left(y\right)\right)}^{2},\\ E(x)= & \frac{1}{K}\sum_{i=1}^{K}x_{i},\;E\left(y\right)=\frac{1}{K}\sum_{i=1}^{K}y_{i}. \end{array}$$

**Figure 8:**
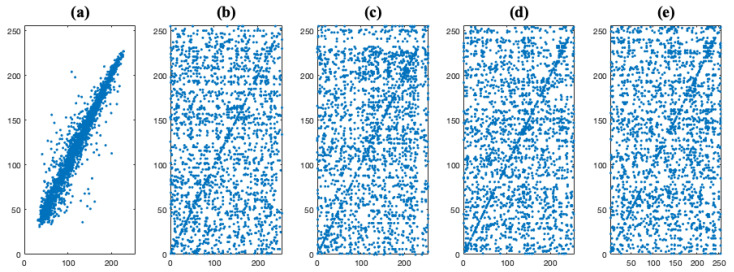
Correlation plot of two adjacent pixels at the horizontal direction for (**a**) the Lena test image and (**b**–**e**) the images $I_{S}$ considering the image encryption systems $E_{1}$, $E_{2}$, $E_{3}$, and $E_{4}$, respectively.

**Figure 9:**
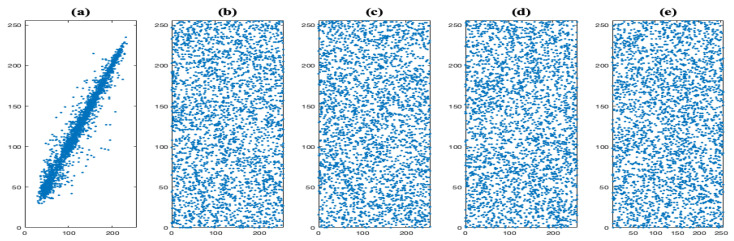
Correlation plot of two adjacent pixels at the horizontal direction for (**a**) the Lena test image and (**b**–**e**) the encrypted images $I_{C}$ with the image encryption systems $E_{1}$, $E_{2}$, $E_{3}$, and $E_{4}$, respectively.

In the last expressions, *x* and *y* represent the corresponding pixels between the two images; *N* is the total number of pixels; and cov(x,y), D(·), and E(·) represent covariance, variance and mean, respectively. Note that we randomly select 5000 pairs of adjacent pixels in each direction from the plain images $I_{O}$ and their respective images $I_{S}$ or $I_{C}$. Then, for each case, we have computed the correlation coefficient of each pair. In Figure 8, the distribution of adjacent pixels at the horizontal direction in the Lena test image and their $I_{S}$ versions is illustrated. The plain image, Figure 8a, presents a strong correlation between adjacent pixels since most of the pixels are on the identity line y=x. As is shown in Figure 8b–e, and after the scrambling stage, independently of the image encryption system, the pixels of images $I_{S}$ are scattered more uniformly, but preserves many pixels on the identity line. This situation is different after the encryption stage, where the pixels of the images $I_{C}$ are scattered very uniformly as is displayed in Figure 9b–e.

The correlation coefficients of the image dataset and the respective $I_{S}$ images with different scrambling processes are listed in Table 2, Table 3, Table 4, Table 5, Table 6 and Table 7, considering the horizontal(h), vertical(v), and diagonal(d) directions. In Table 2 and Table 3 are the results when one operation is applied to the $I_{O}$ images in the scrambling stage the standard and improved ZigZag operation, and the S-box of the $E_{2}$, $E_{3}$, and $E_{4}$ systems, respectively. For the case of the ZigZag transformation, we can observe in both cases a strong correlation in the horizontal direction, but a weak correlation in the rest of the directions, whereas for the $I_{S}$ images obtained with the S-box in the scrambling stage are exhibited just weak correlations. Then, it seems that the application of the S-box in the scrambling stage decreases the correlation coefficients. In addition, we can find that the correlation between adjacent pixels in images $I_{S}$ becomes low when the scrambling process combines a ZigZag transformation with a sorting scrambling algorithm or an S-box, see Table 4 and Table 5. On the other hand, as is shown in Table 6 and Table 7, the correlation coefficients of the encrypted images $I_{C}$ are close to 0, and therefore there is no correlation among the pixels independently of the used encryption scheme, which suggests that such encryption systems can resist statistical attacks.

### 4.4. NPCR and UACI Analysis

In image encryption, the cipher resistance to differential attacks is commonly analyzed with the two measures: the number of pixels changing rate (NPCR) and the unified averaged changed intensity (UACI). Both measures are based on slight changes of two images keeping the key unchanged.

For the original ($I_{O}$) and encrypted ($I_{C}$) images of dimensions M×N, the NPCR make the assessment of the pixel difference between them as follows:

(12) $$\mathrm{NPCR}\left(I_{O},I_{C}\right)=\sum_{i=1}^{M}\sum_{j=1}^{N}\frac{D(i,j)}{M\times N}\times 100,$$

where D(i,j) is calculated as

(13) $$D\left(i,j\right)=\left\{\begin{array}{cc} 0 & I_{O}\left(i,j\right)=I_{C}\left(i,j\right),\\ 1 & I_{O}\left(i,j\right)\neq I_{C}\left(i,j\right). \end{array}\right.$$

In a similar way, the UACI evaluates the mean intensity of differences between the $I_{O}$ and $I_{C}$ images as follows

(14) $$\mathrm{UACI}\left(I_{O},I_{C}\right)=\sum_{i=1}^{M}\sum_{j=1}^{N}\frac{|I_{O}\left(i,j\right)-I_{C}\left(i,j\right)|}{M\times N\times L}\times 100,$$

where *L* is the largest value pixel value of both images. A value of 99% for the NPCR test and a value of 33% for UACI are interpreted as success criteria. As is pointed out in [10,26], for a significance level α, the obtained results are accepted if the NPCR values are greater than the critical NPCR value $N_{\alpha }^{*}$, and the UACI values should be in the critical interval $\left[U_{\alpha }^{*-},U_{\alpha }^{*+}\right]$. Table 8 shows the $N_{\alpha }^{*}$, $U_{\alpha }^{*-}$ and $U_{\alpha }^{*+}$ values for some cases, where, in accordance to the works in [10,26], we also set α=0.05. It seems that the encryption system with two operations at the scrambling stage achieves a better performance, as the critical NPCR value is greater than the encryption system with just one operation at the scrambling stage. To illustrate the evaluation results of the NPCR and UACI, Table 9, Table 10, Table 11, Table 12 and Table 13 present the evaluation results of NPCR and UACI at the scrambling stage and the encryption stage. We can observe a better performance when two operations are considered at the scrambling stage. Such a situation indicates that our conjecture will provide good resistance against differential attacks.

### 4.5. Information Entropy

To measure the randomness of images, the information entropy test was carried out. This test provides us information on the texture of an image and returns a scalar value *H* which is calculated as [27]

(15) $$H\left(s\right)=\sum_{i=0}^{255}p\left(s_{i}\right)\log_{2}\left(\frac{1}{p(s_{i})}\right),$$

where $p(s_{i})$ denotes the probability of the appearance of the symbol $s_{i}$. For each image of 256 gray levels, the more entropy value *H* gets close to the ideal theoretical value of 8, the less possible for attackers to decode encrypted images. Table 14, Table 15 and Table 16 show the numerical entropy values of the $I_{O}$, $I_{S}$, and $I_{C}$ images when the ZigZag transformation, the S-box, and both of them have been applied to images $I_{O}$ in the scrambling stage, respectively. We can observe that the entropy values obtained for the $I_{S}$ images are close or greater than 7, but the $I_{C}$ images present an increment in their entropy values, for all encryption systems, and are close to the ideal value, which also means high resistance to entropy attacks.

### 4.6. Peak Signal to Noise Ratio (PSNR) Analysis

The peak signal to noise ratio (PSNR) has been considered as an objective metric to measure the quality of an image [15]. The PSNR metric is computed as

(16) $$\begin{array}{c} \mathrm{PSNR}\left(I_{O},I_{C}\right)=10\log_{10}\frac{{\left(2^{b}-1\right)}^{2}}{\mathrm{MSE}(I_{O},I_{C})}, \end{array}$$

(17) $$\begin{array}{c} \mathrm{MSE}\left(I_{O},I_{C}\right)=\frac{1}{MN}\sum_{i=1}^{M}\sum_{j=1}^{N}{\left[I_{O}\left(i,j\right)-I_{C}\left(i,j\right)\right]}^{2}, \end{array}$$

where $I_{O}$ is the original image, $I_{C}$ is the encrypted image, and *b* is the number of bits required to represent each pixel of the images, which is equal to 8. The mean squared error, which is denoted by MSE, is defined by Equation (17), where MN is the size of the images, whereas (i,j) corresponds to the coordinates of the pixel. The value of the PSNR represents the similarity between the images $I_{O}$ and $I_{C}$, where the higher the value of PSNR, the lesser error or greater similarity between them [7,15,16].

Table 17 shows the results of the PSNR between the original images $I_{O}$ with their respective images at the scrambling and encryption stages, $I_{S}$ and $I_{C}$, respectively, when two operations are considered at the scrambling stage. It is observed that the obtained values of PSNR are low for all the encrypted images, hence they also show that our proposals are good.

### 4.7. 2D-DFA Metric

To carry out the scaling analysis of the different encryption systems, we apply the 2D-DFA to the $I_{O}$, $I_{S}$, and $I_{C}$ images when the scrambling stage considers one or two operations. Table 18 and Table 19 provide the scaling exponents when the standard or improved version of the ZigZag operation or an S-box is applied to $I_{O}$ images, respectively. For this metric, the scaling exponents present similar values for $I_{S}$ and $I_{C}$ versions, where the scaling exponent has a lower value compared to the obtained of the $I_{O}$ images, and some information of the original image may be revealed.

On the other hand, Table 20 shows the results of the scaling analysis for all encryption systems considered in this work with a scrambling stage with two operations. For this metric, the values of the scaling exponents of the $I_{S}$ images are lower than the obtained for the $I_{O}$ images. Even more, the scaling exponent values of the encrypted images are close to 1, which means that the analyzed information presents a persistent behavior, and according to the work in [15], the encrypted images do not reveal any piece of information that can allow to distinguish the original images.

**Figure 10:**
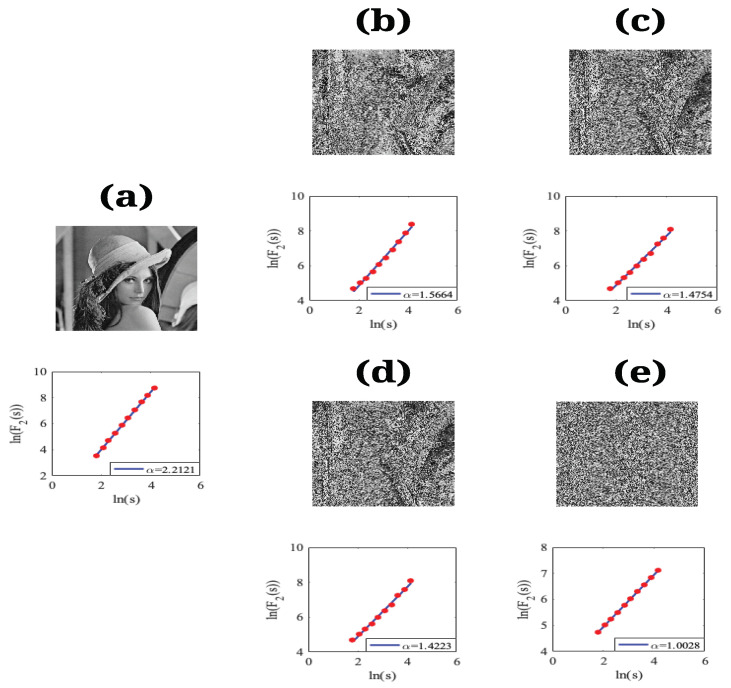
(**a**) The Lena test image and its respective scaling analysis. (**b**,**c**) The $I_{S}$ and $I_{C}$ images with their respective scaling analysis, where the S-box of the $E_{2}$ system is considered in the scrambling stage. (**d**,**e**) The $I_{S}$ and $I_{C}$ images with their respective scaling analysis, where the complete scrambling stage in the $E_{2}$ system is considered.

As an example, Figure 10 shows the results of the performance of the 2D-DFA of the Lena test image. In Figure 10a are the $I_{O}$ image and its respective scaling exponent α≈2.2121; Figure 10b,c corresponds to $I_{S}$ and $I_{C}$ when the S-box of $E_{2}$ is considered in the scrambling stage with scaling exponents α≈1.5664 and α≈1.4754, respectively. In this case, we can observe acceptable levels of confusion in the visual form of the encrypted image, but we cannot achieve a complete unintelligible form. On the other hand, Figure 10d,e corresponds to $I_{S}$ and $I_{C}$ when the complete scrambling stage in the $E_{2}$ system with scaling exponents α≈1.4223 and α≈1.0028, respectively. For this case, we notice that the scaling exponent α is close to unity, and the encrypted image does not reveal information. Therefore, we consider that this 2D-DFA method is an efficient tool to describe this kind of image in terms of the scaling exponent values, which are in agreement with those obtained in [15].

## 5. Discussion

Despite that the main architecture of the encryption system as well as the main operations that we consider are well known from literature, only a few attempts have been made to establish how many operations or transformations are required in an image encryption system, or if an evaluation of internal stages of the complete encryption process may be helpful in the design of image encryption systems.

**Figure 11:**
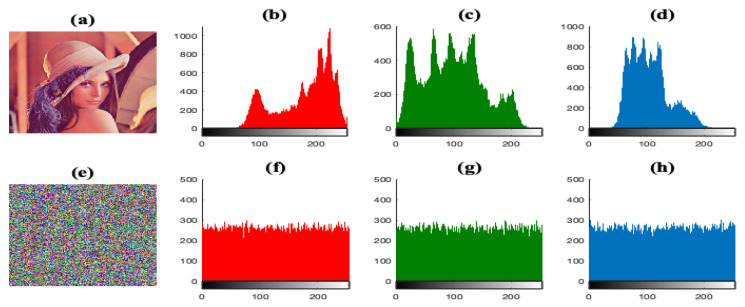
Histogram analysis for the color Lena test image. (**a**) The plain-image $I_{O}$. (**b**–**d**) Histograms for red, green and blue channels, respectively. (**e**) The encrypted Lena image considering the image encryption system $E_{1}$. (**f**–**h**) The corresponding histograms for red, green and blue channels of the encrypted image (e).

There are some missing tests to assess the performance of our proposals, and with the aim to carry out a performance comparison of some existing works with our proposals, we apply the encryption system $E_{2}$ for a color Lena image (256×256). In Figure 11, we illustrate the histograms for the Lena test image, its encrypted version, and their respective histograms for each color intensity level. From the figures, one can see that the histograms of the encrypted versions are uniformly distributed and significantly different from the respective histograms of the original image, which indicates that it would be difficult for the attacker to decipher the image content.

**Figure 12:**
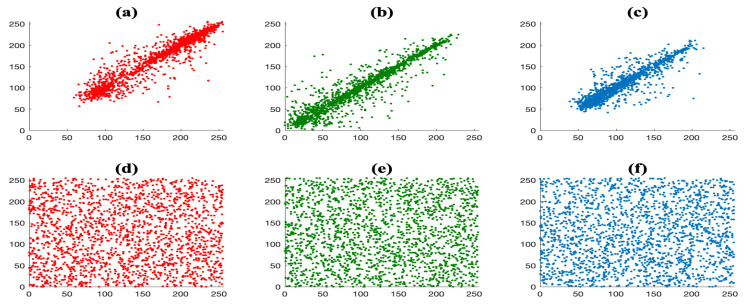
Correlation plot of two adjacent pixels for the color Lena test image (**top**) and its encrypted version (**bottom**), using $E_{2}$, at the horizontal (**first column**), vertical (**second column**), and diagonal (**third column**) direction.

In Figure 12, the distribution of adjacent pixels at the horizontal, vertical and diagonal directions for the color Lena image (top row) and its encrypted version (bottom row) is shown. Figure 12a–c illustrate a strong correlation between adjacent pixels along the three directions, whereas there is no correlation among the pixels for the encrypted version, see Figure 12d–f.

**Figure 13:**
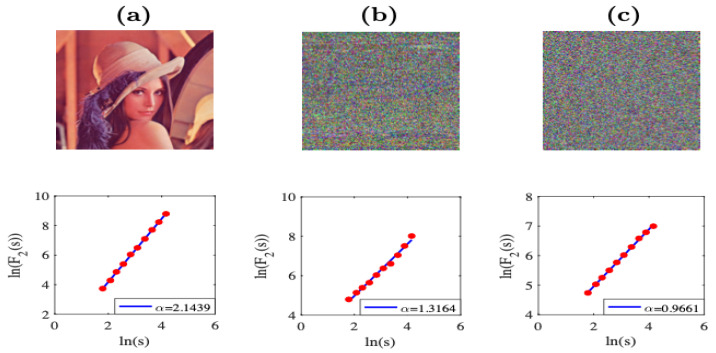
(**a**) The color Lena test image, (**b**) the $I_{S}$, and (**c**) $I_{C}$ images, with their respective scaling analysis when the complete scrambling stage in the $E_{2}$ system is considered.

Figure 13 shows the scaling results when the 2D-DFA is applied to the color Lena image in the $E_{2}$ system. The results are very similar to the case of the grayscale Lena image presented in Figure 10, as the scaling exponent α is closer to unity, the encrypted image does not reveal information.

Furthermore, Table 21 contains the results of the comparison of our proposals with other methods proposed by Li et al. [11], Ahmad et al. [12], and Ramasamy et al. [7], where the correlation analysis, entropy, NPCR, UACI, and PSNR were considered. The bold values shown in Table 21 indicate that our results are quite comparable with the other methods. Moreover, Table 22 shows a speed analysis test, where the algorithms in [7,13,28,29] were considered. The results show that our proposals are computationally efficient.

In this context, it is observed that through certain tests we analyzed the impact of considering two operations in the scrambling block in some image encryption systems that are based on hyperchaotic systems. Our main observations are as follows: (a) To consider more than one operation or transformation in the scrambling stage present an increment in security; (b) the use of a hyperchaotic dynamical system to remove pixel correlation and the key generation is desirable to exploit the chaotic characteristics in image encryption algorithms; and (c) although some standard tests make a good evaluation, the scaling analysis outperforms as an objective metric.

However, we still cannot establish how many operations are optimal in the scrambling stage without affect substantially the execution time; which operations will provide a better performance, or which is the best hyperchaotic system. In addition, a larger number of images must be considered in the analysis.

## 6. Conclusions

In this work, we have used some statistical tests and some quality metrics to analyze a known encryption system and some variants, which are based on hyperchaotic dynamical systems. In those variants, such hyperchaotic systems are used to generate S-boxes as well as the key generation in the encryption systems. Although many similar encryption systems have been proposed, little attention has been paid to evaluate some internal encryption stages or processes. Our results show that the encryption systems improve the performance when the scrambling block includes two operations or transformations. This could help in the design or implementation of this kind of encryption system.

In addition, the 2D-DFA method seems to be more sensitive than some statistical tests to characterize images in different stages of the encryption process. In our opinion, this is an efficient metric to indicate if the encrypted images may reveal or not any image information. Thus, it is suggested that such a scaling exponent can be used as an objective metric of the quality in different encryption schemes. In fact, a good image encryption method should obtain a scaling exponent close to unity independently of what encryption system is used. As is pointed out in [15], one cannot fully guarantee so far that an encrypted image with such values of the scaling exponent is absolutely immune to any type of attack, but we consider that this tool with the help of other metrics can provide enhanced security to encryption systems.

Although many image encryption algorithms based on chaotic dynamical systems have been proposed, we believe that our approach can be helpful in the design and analysis of such systems, as it shows a performance that is comparable with other similar systems. From the security point of view, the results show that the scaling analysis used here can be an appropriate measure to assess the quality of encryption methods. In the future, we aim to evaluate different kinds of operations in the scrambling stage to increase the security without affecting drastically the processing time. We are also considering to carry out an extensive examination of which chaotic or hyperchaotic dynamical systems may improve the protection of the image content, as well as to make a better assessment based on a larger set of images.

## Figures and Tables

**Table 1:** *p*-values of the hypothesis test for the encrypted images.

Image	*p*-Values			
	$E_{1}$	$E_{2}$	$E_{3}$	$E_{4}$
1	0.3187	0.1375	0.7522	0.3187
2	0.2523	0.2315	0.5076	0.3419
3	0.0615	0.7384	0.0052	0.1463
4	0.6579	0.2443	0.9445	0.4398
5	0.3718	0.5787	0.5150	0.3920
6	0.7848	0.9676	0.2627	0.8596

**Table 2:** Correlation coefficients between adjacent pixels of plain images and their $I_{S}$ images considering the standard and improved ZigZag transformation in the scrambling stage.

Correlation Coefficients									
Image	$I_{o}$			Standard ZigZag Operation			Improved ZigZag Operation		
	h	v	d	h	v	d	h	v	d
1	0.9838	0.9858	0.9743	0.9747	−0.0549	−0.0520	0.9688	0.5624	0.5627
2	0.9848	0.9895	0.9798	0.9670	0.0328	0.0377	0.9647	0.3275	0.3320
3	0.9710	0.9791	0.9605	0.9412	0.1039	0.0979	0.9387	0.3542	0.3603
4	0.9727	0.9752	0.9629	0.9343	0.2256	0.2149	0.9319	0.3555	0.3564
5	0.9942	0.9959	0.9923	0.9937	0.0497	0.0495	0.9862	0.4799	0.4798
6	0.9477	0.9317	0.9078	0.8662	0.1003	0.1221	0.8579	0.3117	0.2972

**Table 3:** Correlation coefficients between adjacent pixels of plain images and their $I_{S}$ images obtained with the S-box of the $E_{2}$, $E_{3}$ and $E_{4}$ systems in the scrambling stage.

Correlation Coefficients									
Image	$E_{2}$			$E_{3}$			$E_{4}$		
	h	v	d	h	v	d	h	v	d
1	0.2330	0.2311	0.1852	0.3580	0.3275	0.2483	0.1984	0.2494	0.1758
2	0.1442	0.1348	0.0636	0.1497	0.1945	0.1442	0.0862	0.1019	0.0618
3	0.0585	0.0917	0.0531	0.1387	0.1353	0.0740	0.0871	0.0596	0.0426
4	0.1095	0.1383	0.0748	0.1709	0.1870	0.1522	0.0687	0.0532	0.0418
5	0.1104	0.1384	0.1123	0.2002	0.2715	0.1695	0.1470	0.1234	0.0676
6	0.0742	0.0462	0.0474	0.0889	0.1113	0.0707	0.0280	0.0489	0.0444

**Table 4:** Correlation coefficients between adjacent pixels of the $I_{S}$ images considering the $E_{1}$ and $E_{2}$ systems.

Correlation Coefficients						
Image	$E_{1}$			$E_{2}$		
	h	v	d	h	v	d
1	0.0153	−0.0309	0.0014	0.2095	0.2165	0.1432
2	0.0188	0.0297	−0.0003	0.0122	0.0120	0.0979
3	0.0109	−0.0025	0.0004	0.0752	0.0825	0.0513
4	0.0016	0.0588	0.0130	0.0100	0.0701	0.0980
5	0.0065	0.0152	0.0182	0.0143	0.0012	0.0092
6	−0.0001	−0.0016	−0.0661	0.0031	0.0032	0.0023

**Table 5:** Correlation coefficients between adjacent pixels of the $I_{S}$ images considering the $E_{3}$ and $E_{4}$ systems.

Correlation Coefficients						
Image	$E_{3}$			$E_{4}$		
	h	v	d	h	v	d
1	0.3499	0.3146	0.2588	0.2094	0.2248	0.1455
2	0.1608	0.1931	0.1327	0.0822	0.0874	0.0887
3	0.0143	0.0134	0.0129	0.0952	0.0977	0.0543
4	0.0162	0.0212	0.0151	0.0726	0.0517	0.0516
5	0.0021	0.0023	0.0019	0.1134	0.1338	0.1156
6	0.0812	0.0043	0.0690	0.0578	0.0542	0.0158

**Table 6:** Correlation coefficients between adjacent pixels of the $I_{C}$ images considering the $E_{1}$ and $E_{2}$ systems.

Correlation Coefficients						
Image	$E_{1}$			$E_{2}$		
	h	v	d	h	v	d
1	0.0690	0.0034	0.0261	0.0029	−0.0019	−0.0126
2	0.0727	−0.0198	−0.484	0.0029	−0.0019	−0.0126
3	−0.0087	−0.0078	0.0239	0.0698	0.0729	0.0792
4	−0.0035	0.0096	−0.0190	0.0117	−0.0225	0.0156
5	0.0511	−0.050	−0.0039	0.0183	0.0092	−0.0168
6	−0.0058	−0.0050	0.0452	0.0044	0.0211	0.0159

**Table 7:** Correlation coefficients between adjacent pixels of the $I_{C}$ images considering the $E_{3}$ and $E_{4}$ systems.

Correlation Coefficients						
Image	$E_{3}$			$E_{4}$		
	h	v	d	h	v	d
1	0.0036	0.0048	0.0152	−0.0050	0.0006	0.0015
2	0.0036	0.0048	0.0152	−0.0156	−0.0115	0.0189
3	0.1399	0.1293	0.0976	0.0261	−0.0014	0.0288
4	0.0068	−0.0062	−0.0018	−0.0018	0.0250	−0.0057
5	−0.0193	−0.0031	−0.0103	0.0178	−0.0139	0.0061
6	0.0015	0.0016	−0.0109	−0.0028	0.0104	−0.0143

**Table 8:** Expected NPCR (%) and UACI (%) values for some cases when the standard ZigZag (S-ZZ) and improved ZigZag (I-ZZ) transformation are applied to images $I_{O}$ in the scrambling and encryption stages.

Image →	$I_{S}$			$I_{C}$		
**System**↓	$N_{\alpha }^{*}$	$U_{\alpha }^{*-}$	$U_{\alpha }^{*+}$	$N_{\alpha }^{*}$	$U_{\alpha }^{*-}$	$U_{\alpha }^{*+}$
$E_{1}$ with I-ZZ	97.5023	32.1023	32.9938	98.1233	33.3312	33.6310
$E_{2}$ with S-box	97.9002	31.1025	31.9533	98.2313	33.1133	33.7521
$E_{2}$ with S-ZZ and S-box	99.3312	33.2815	33.5731	99.6135	33.3328	33.5451

**Table 9:** NPCR (%) and UACI (%) values when the standard ZigZag (S-ZZ) and improved ZigZag (I-ZZ) transformation are applied to images $I_{O}$ in the scrambling and encryption stages.

	$I_{S}$				$I_{C}$			
Image	S-ZZ		I-ZZ		S-ZZ		I-ZZ	
	NPCR	UACI	NPCR	UACI	NPCR	UACI	NPCR	UACI
1	97.5672	33.3830	97.0600	31.2290	98.6108	33.3330	97.0137	32.2630
2	97.6622	32.9965	98.1769	31.9929	98.7830	32.2187	97.1992	32.5631
3	97.4531	32.1238	97.9945	31.9995	98.8612	33.0953	98.0945	32.9752
4	97.9954	31.9549	97.4301	31.9437	98.0167	33.3316	98.9012	33.2139
5	97.9128	32.9981	98.1956	32.2190	98.5621	33.4319	98.1605	33.4691
6	97.4182	32.4794	97.3981	32.2964	98.1598	33.4189	98.9158	33.4498
Pass	5	5	6	5	5	4	5	5
Mean	97.6681	32.6559	97.7092	31.9466	98.4989	33.1377	98.0474	32.9940
Std	0.0571	0.3134	0.2265	0.1432	0.1151	0.2175	0.6572	0.2404

**Table 10:** NPCR (%) and UACI (%) values considering $I_{O}$ and $I_{S}$ images when the S-box of the $E_{2}$, $E_{3}$ and $E_{4}$ systems are applied to images $I_{O}$ in the scrambling stage.

	$I_{S}$					
Image	$E_{2}$		$E_{3}$		$E_{4}$	
	NPCR	UACI	NPCR	UACI	NPCR	UACI
1	97.9124	31.9252	97.0467	32.3768	96.9961	32.4314
2	98.0496	31.2461	97.0459	32.4592	97.1198	32.9832
3	97.9047	31.9010	97.8830	32.1674	97.9179	32.9174
4	98.3955	31.2061	97.7819	31.0194	97.5991	31.0173
5	97.8728	31.2187	97.1298	32.2109	97.0652	32.2487
6	98.1807	31.2205	97.9612	31.0175	97.1921	31.0147
Pass	6	6	5	4	5	4
Mean	98.0526	31.4529	97.4747	31.8752	97.3150	32.1021
Std	0.0415	0.1272	0.1967	0.4517	0.1323	0.7860

**Table 11:** NPCR (%) and UACI (%) values considering the $I_{O}$ and $I_{C}$ images when the S-box of the $E_{2}$, $E_{3}$, and $E_{4}$ systems are applied to images $I_{O}$ in the scrambling stage.

	$I_{S}$					
Image	$E_{2}$		$E_{3}$		$E_{4}$	
	NPCR	UACI	NPCR	UACI	NPCR	UACI
1	99.0783	33.1338	98.2983	32.9927	99.0485	32.9832
2	98.9916	33.1859	98.9630	32.7950	99.7391	33.3861
3	99.5842	33.4997	99.4598	33.0937	98.9937	33.2487
4	98.4461	33.2643	99.5293	33.2197	98.3671	33.2201
5	98.1845	33.3432	98.9932	33.3141	98.6825	33.2826
6	98.2901	32.3379	98.2017	33.3357	98.9951	33.3261
Pass	5	6	5	5	5	5
Mean	98.7624	33.1274	98.9075	33.1251	98.9710	33.2411
Std	0.2969	0.1661	0.3142	0.0433	0.2090	0.0193

**Table 12:** NPCR (%) and UACI (%) values considering $I_{O}$ and $I_{S}$ images with the $E_{1}-E_{4}$ systems in the scrambling stage with two operations.

	Scrambling Block							
Image	NPCR				UACI			
	$E_{1}$	$E_{2}$	$E_{3}$	$E_{4}$	$E_{1}$	$E_{2}$	$E_{3}$	$E_{4}$
1	98.9993	99.5691	99.6881	99.1727	33.4662	33.2931	33.4621	33.4638
2	99.3956	99.6142	99.6129	99.4328	33.3687	33.4637	33.4674	33.4643
3	99.2137	99.6344	99.6017	99.3449	33.4431	33.4631	33.4538	33.4459
4	99.4429	99.6147	99.6045	99.6327	33.4537	33.4638	33.4625	33.4452
5	99.4414	99.6134	99.6827	99.6157	33.4238	33.4545	33.4545	33.4637
6	99.4215	99.6135	99.6122	99.6020	33.4632	33.4623	33.4632	33.4628
Pass	6	6	6	6	6	6	6	6
Mean	99.3190	99.6098	99.6336	99.4667	33.4364	33.4334	33.4605	33.4576
Std	0.0320	0.0004	0.0016	0.0340	0.0013	0.0047	0.0025	0.0084

**Table 13:** NPCR (%) and UACI (%) values considering $I_{O}$ and $I_{C}$ images with the $E_{1}-E_{4}$ systems when two operations are considered in the scrambling stage.

	Encryption Block							
Image	NPCR				UACI			
	$E_{1}$	$E_{2}$	$E_{3}$	$E_{4}$	$E_{1}$	$E_{2}$	$E_{3}$	$E_{4}$
1	99.6112	99.6226	99.9083	99.6028	33.4926	33.4748	33.4843	33.4838
2	99.5932	99.6633	99.6825	99.6033	33.4693	33.4683	33.4683	33.4782
3	99.6135	99.6383	99.6838	99.6139	33.4739	33.4874	33.4635	33.4632
4	99.6133	99.6253	99.6335	99.6873	33.4843	33.4724	33.4639	33.4639
5	99.6332	99.7823	99.7172	99.7643	33.4934	33.4891	33.4718	33.4763
6	99.6123	99.6298	99.6382	99.6273	33.4793	33.4636	33.4693	33.4697
Pass	6	6	6	6	6	6	6	6
Mean	99.6127	99.6552	99.7105	99.6498	33.4821	33.4759	33.4701	33.4725
Std	0.0016	0.0039	0.0103	0.0041	0.0937	0.0012	0.0054	0.0063

**Table 14:** The comparison of information entropies for the $I_{S}$ and $I_{C}$ images when the standard ZigZag (S-ZZ) and improved ZigZag (I-ZZ) transformation are applied to images $I_{O}$ in the scrambling stage.

Entropy					
Image		$I_{S}$		$I_{C}$	
	$I_{O}$	S-ZZ	I-ZZ	S-ZZ	I-ZZ
1	7.0478	7.0479	7.0477	7.9989	7.9980
2	7.4451	7.4452	7.4449	7.9977	7.9975
3	7.2367	7.2367	7.2368	7.9966	7.9964
4	6.9542	6.9544	6.9541	7.9965	7.9953
5	7.2757	7.2760	7.2765	7.9974	7.9983
6	7.2925	7.2930	7.2921	7.9990	7.9990

**Table 15:** The comparison of information entropies for the $I_{S}$ and $I_{C}$ images when the S-box of the $E_{2}$, $E_{3}$, and $E_{4}$ systems is applied to images $I_{O}$ in the scrambling stage.

Entropy						
Image	$I_{S}$			$I_{C}$		
	$E_{2}$	$E_{3}$	$E_{4}$	$E_{2}$	$E_{3}$	$E_{4}$
1	7.0477	7.0480	7.0478	7.9980	7.9984	7.9983
2	7.4459	7.4465	7.4460	7.9986	7.9984	7.9988
3	7.2370	7.2374	7.2367	7.9990	7.9991	7.9991
4	6.9550	6.9548	6.9545	7.9991	7.9991	7.9991
5	7.2740	7.2743	7.2740	7.9983	7.9987	7.9980
6	7.2935	7.2930	7.2929	7.9991	7.9991	7.9989

**Table 16:** The comparison of information entropies for the $I_{S}$ and $I_{C}$ images when the complete scrambling stage is applied to images $I_{O}$.

	Entropy							
Image	$I_{S}$				$I_{C}$			
	$E_{1}$	$E_{2}$	$E_{3}$	$E_{4}$	$E_{1}$	$E_{2}$	$E_{3}$	$E_{4}$
1	7.0478	7.0477	7.0477	7.0464	7.9993	7.9993	7.9993	7.9993
2	7.4451	7.4451	7.4451	7.4451	7.9993	7.9993	7.9993	7.9993
3	7.2367	7.2367	7.2367	7.2367	7.9992	7.9993	7.9994	7.9993
4	6.9542	6.9542	6.9542	6.9542	7.9993	7.9993	7.9993	7.9994
5	7.2757	7.2737	7.2737	7.2757	7.9992	7.9992	7.9993	7.9994
6	7.2925	7.2925	7.2925	7.2925	7.9993	7.9993	7.9993	7.9994

**Table 17:** PNSR values in $I_{C}$ considering the complete scrambling.

	PSNR Values							
Image	$I_{S}$				$I_{C}$			
	$E_{1}$	$E_{2}$	$E_{3}$	$E_{4}$	$E_{1}$	$E_{2}$	$E_{3}$	$E_{4}$
1	13.1462	15.7231	13.0126	13.0480	7.8623	7.4127	7.9827	7.4568
2	12.1596	13.0690	12.5056	12.1596	8.3917	8.4818	8.6578	8.2682
3	12.4326	13.2715	13.2670	11.2021	8.8176	8.8086	8.8526	8.8264
4	13.3398	12.4504	12.4812	13.5120	7.9042	7.9827	7.8129	7.6559
5	11.3313	11.4401	11.0419	11.4757	8.9271	8.8597	8.8045	8.7528
6	13.8149	13.7376	13.6122	13.3356	9.4522	9.4782	9.4529	9.2740

**Table 18:** The comparison of information of the scaling exponents obtained from applying the 2D-DFA scheme to the $I_{O}$, $I_{S}$, and $I_{C}$ images, when the standard ZigZag (S-ZZ) and improved ZigZag (I-ZZ) transformation are applied to images $I_{O}$ in the scrambling stage.

	α Exponents				
Image		$I_{S}$		$I_{C}$	
	$I_{O}$	S-ZZ	I-ZZ	S-ZZ	I-ZZ
1	2.1990	1.9504	1.7666	1.8956	1.8603
2	2.2121	1.8130	1.6232	1.8845	1.8556
3	2.2851	1.8130	1.6389	1.6329	1.8594
4	2.1970	1.8808	1.5421	1.5421	1.8063
5	2.5659	1.8495	1.8152	1.7881	1.6562
6	1.9218	1.7336	1.8091	1.9323	1.8921

**Table 19:** The comparison of information of the scaling exponents obtained from applying the 2D-DFA scheme to the $I_{S}$ and $I_{C}$ images, when the S-box of the $E_{2}$, $E_{3}$, and $E_{4}$ systems is applied to images $I_{O}$ in the scrambling stage.

	α Exponents					
Image	$I_{S}$			$I_{C}$		
	$E_{2}$	$E_{3}$	$E_{4}$	$E_{2}$	$E_{3}$	$E_{4}$
1	1.5664	1.6356	1.5737	1.7754	1.7941	1.7881
2	1.4223	1.5746	1.4929	1.5667	1.4651	1.6956
3	1.3669	1.5002	1.4416	1.6796	1.7534	1.7598
4	1.5422	1.5627	1.3373	1.6988	1.6793	1.7018
5	1.5152	1.6566	1.5156	1.5583	1.5868	1.6039
6	1.1891	1.2609	1.2655	1.5624	1.5617	1.6117

**Table 20:** The comparison of information of the scaling exponents obtained from applying the 2D-DFA scheme to the $I_{S}$ and $I_{C}$ images when the complete scrambling stage is applied to images $I_{O}$.

	α Exponents							
Image	$I_{S}$				$I_{C}$			
	$E_{1}$	$E_{2}$	$E_{3}$	$E_{4}$	$E_{1}$	$E_{2}$	$E_{3}$	$E_{4}$
1	1.5351	1.5464	1.6356	1.5737	1.1426	0.9999	1.0018	1.0481
2	1.5444	1.4223	1.5746	1.4929	1.1545	1.0028	1.0072	1.0556
3	1.5528	1.3669	1.5002	1.4416	1.1394	1.0030	1.0016	1.0380
4	1.5652	1.5422	1.5627	1.3373	1.1346	1.0224	0.9948	1.0648
5	1.5438	1.5152	1.6566	1.5156	1.1386	0.9955	1.0318	1.0393
6	1.5616	1.1891	1.2609	1.2665	1.1222	0.9926	1.0141	1.0631

**Table 21:** Performance evaluation and comparison with other methods considering as original image the Lena color image.

Measure	[11]	[12]	[7]	Proposed		
				$E_{1}$ [3]	$E_{2}$	$E_{3}$
Horizontal correlation	0.0327	**0.0026**	−0.0237	0.0219	−0.0037	0.0091
Vertical correlation	0.0219	−0.0038	−0.0178	0.0128	−0.0278	**0.0029**
Diagonal correlation	0.0180	−0.0062	−0.0284	−0.0059	**0.0041**	−0.0158
Entropy	7.9993	7.9832	**7.9995**	7.9990	7.9990	7.9990
NPCR	n/a	**0.9966**	0.9962	0.9961	**0.9966**	0.9960
UACI	n/a	0.3362	**0.3358**	0.3345	0.3346	0.3346
PSNR	n/a	8.3656	6.7494	**4.7465**	4.7961	4.8101

**Table 22:** Comparison of computational time for the proposed algorithms.

	Algorithms							
	[28]	[29]	[13]	[7]	Proposed			
					$E_{1}$ [3]	$E_{2}$	$E_{3}$	$E_{4}$
Time (seconds)	2.414	2.169	2.386	2.087	2.055	1.913	1.925	1.992

## Data Availability

Not applicable.

## Abbreviations

S-ZZ	Standard ZigZag transformation
I-ZZ	Improved ZigZag transformation
S-box	Substitution box
2D-DFA	Two-dimensional detrended fluctuation analysis

## Appendix A. S-Boxes Based on the Hyperchaotic Systems

Table A1 and Table A2 illustrate, in the conventional representation format, the elements of the two S-boxes considered in this work when the hyperchaotic Lorenz system and the hyperchaotic Chen system are considered, respectively.

**Table A1:** Elements of the S-box based on the hyperchaotic Lorenz system (1) in the form of a 16×16 matrix.

	0	1	2	3	4	5	6	7	8	9	A	B	C	D	E	F
**0**	212	188	234	104	49	245	186	249	30	42	5	187	93	254	144	113
**1**	158	143	140	251	55	203	100	236	125	123	169	58	218	38	160	18
**2**	173	0	127	248	126	255	137	46	240	130	134	48	230	141	19	180
**3**	153	159	163	164	131	166	165	14	238	44	229	146	112	71	129	106
**4**	109	92	210	75	21	214	150	77	27	228	11	111	51	3	177	148
**5**	15	96	207	168	182	244	89	50	170	156	105	47	246	190	135	197
**6**	31	124	250	237	6	219	178	205	199	61	213	139	133	67	16	40
**7**	152	82	162	41	9	62	193	220	107	138	198	84	208	69	116	78
**8**	192	102	1	154	224	174	151	54	227	231	43	221	36	52	217	171
**9**	97	59	56	194	2	120	88	4	209	60	64	79	80	145	26	66
**A**	34	37	101	10	28	202	157	53	216	103	181	29	235	87	115	233
**B**	83	13	247	232	119	122	32	242	172	7	95	90	17	86	184	81
**C**	68	149	74	25	12	147	211	200	94	252	155	195	223	23	33	206
**D**	191	185	179	117	243	22	39	57	85	114	136	24	176	108	63	225
**E**	196	98	99	8	204	45	72	65	76	121	128	118	132	142	161	110
**F**	175	183	222	201	215	70	239	241	253	167	20	73	35	91	226	189

**Table A2:** Elements of the S-box based on the hyperchaotic Chen system (2) in the form of a 16×16 matrix.

	0	1	2	3	4	5	6	7	8	9	A	B	C	D	E	F
**0**	41	38	98	28	75	233	51	192	12	7	4	245	136	252	181	247
**1**	84	71	123	237	34	20	235	234	240	211	191	236	194	176	239	117
**2**	242	83	80	60	36	24	5	14	215	179	154	31	118	116	95	56
**3**	55	77	88	204	110	202	130	106	139	167	183	197	226	29	13	151
**4**	207	112	153	137	114	91	0	230	190	246	238	128	108	82	131	48
**5**	161	62	45	241	42	70	209	138	166	145	133	127	121	115	66	157
**6**	200	99	19	59	97	250	163	206	216	208	61	199	193	227	203	249
**7**	210	225	78	105	135	16	185	223	49	64	40	147	125	142	35	129
**8**	124	170	165	177	186	140	205	212	221	86	232	119	6	222	214	43
**9**	196	189	175	173	180	255	44	159	224	104	63	76	155	219	18	8
**A**	3	94	141	182	164	26	132	198	143	47	54	22	21	9	25	218
**B**	111	172	150	251	27	113	101	73	195	228	30	33	74	248	229	126
**C**	187	254	156	217	79	11	120	107	96	72	1	68	57	52	89	39
**D**	32	23	168	17	213	244	2	10	243	50	231	220	93	109	122	134
**E**	67	188	184	178	85	201	92	174	171	253	169	15	162	160	158	103
**F**	37	102	46	100	152	53	149	58	148	90	87	81	146	144	69	65
